# Lack of Evidence for Ribavirin Treatment of Lassa Fever in Systematic Review of Published and Unpublished Studies^1^

**DOI:** 10.3201/eid2808.211787

**Published:** 2022-08

**Authors:** Hung-Yuan Cheng, Clare E. French, Alex P. Salam, Sarah Dawson, Alexandra McAleenan, Luke A. McGuinness, Jelena Savović, Peter W. Horby, Jonathan A.C. Sterne

**Affiliations:** Population Health Sciences, University of Bristol, Bristol, UK (H.-Y. Cheng, C.E. French, S. Dawson, A. McAleenan, L.A. McGuinness, J. Savović, J.A.C. Sterne);; National Institute for Health and Care Research (NIHR) Health Protection Research Unit in Behavioural Science and Evaluation, University of Bristol, Bristol (C.E. French);; United Kingdom Public Health Rapid Support Team, London, UK (A.P. Salam);; Pandemic Sciences Centre, University of Oxford, Oxford, UK (A.P. Salam, P.W. Horby);; NIHR Applied Research Collaboration West, Bristol (J. Savović);; International Severe Acute Respiratory and Emerging Infections Consortium, Oxford (P. Horby);; NIHR Bristol Biomedical Research Centre, Bristol (J.A.C. Sterne);; Health Data Research UK South West, Bristol (J.A.C. Sterne)

**Keywords:** Lassa fever, viruses, ribavirin, systematic review, bias, observational study

## Abstract

Ribavirin has been used widely to treat Lassa fever in West Africa since the 1980s. However, few studies have systematically appraised the evidence for its use. We conducted a systematic review of published and unpublished literature retrieved from electronic databases and gray literature from inception to March 8, 2022. We identified 13 studies of the comparative effectiveness of ribavirin versus no ribavirin treatment on mortality outcomes, including unpublished data from a study in Sierra Leone provided through a US Freedom of Information Act request. Although ribavirin was associated with decreased mortality rates, results of these studies were at critical or serious risk for bias when appraised using the ROBINS-I tool. Important risks for bias related to lack of control for confounders, immortal time bias, and missing outcome data. Robust evidence supporting the use of ribavirin in Lassa fever is lacking. Well-conducted clinical trials to elucidate the effectiveness of ribavirin for Lassa fever are needed.

Lassa virus infection, first described in 1962, is a viral hemorrhagic fever (*1*). It is a substantial public health burden, causing an estimated 100,000–200,000 cases each year, mainly in West Africa (*2*,*3*). Many cases are mild or asymptomatic and are not formally diagnosed (*4*). The nonspecific clinical manifestation makes Lassa fever difficult to recognize on clinical grounds alone, especially in the early phases. The case-fatality rate is estimated to be 10%–20% in hospitalized patients (*5*,*6*) but increases sharply during outbreaks (*7*). No vaccine is available, but studies examining recombinant vaccinia virus in animals have entered the preclinical phase, and a DNA vaccine has entered a phase I trial in humans (*8*–*10*). Lassa virus is part of the US Centers for Disease Control and Prevention’s list of category A Select Agents and is considered a priority pathogen by the World Health Organization (WHO) because of its epidemic potential, its severity, lack of available vaccines, and, most important, limited therapeutic options.

The most influential study of the efficacy of ribavirin in treatment of Lassa fever, published in 1986, reported that administration of intravenous ribavirin within the first 6 days of illness decreased mortality rates from severe Lassa fever from 55% to 5% (*11*). These findings have underpinned the widespread use of, and unequivocal recommendations for, ribavirin for treatment of Lassa fever. Several retrospective observational studies document the use of ribavirin and describe lower case-fatality rates in patients treated with ribavirin (*12*–*17*). However, potential biases in those results make it difficult to evaluate the effectiveness of ribavirin in clinical practice. Recent unpublished results obtained through the US Freedom of Information Act, and secondary analysis of these results, weaken the case for use of ribavirin (*18*). Therefore, we undertook a systematic review of published and unpublished study results, which we appraised by using a state-of-the-art risk for bias tool (*19*), to evaluate ribavirin for treating Lassa fever.

## Methods

This review follows the guidelines of Preferred Reporting Items for Systematic Reviews and Meta-Analyses (PRISMA) (*20*) (Appendix Table 1). A protocol is registered on the International Prospective Register of Systematic Reviews (PROSPERO 2019 CRD42019141818) (https://www.crd.york.ac.uk/prospero).

We conducted a comprehensive search of multiple bibliographic databases from inception to March 8, 2022: Ovid Medline, Ovid Embase, Central Register of Controlled Trials, BIOSIS, WHO Global Index Medicus, and Web of Science (including Science Citation Index Expanded and Conference Proceedings Citation Index-Science). We also searched the WHO International Clinical Trials Registry Platform, ClinicalTrials.gov, and Pan African Clinical Trial Registry databases to identify relevant reports. We searched the keywords “Lassa” and “ribavirin” within Google.com and the WHO website to retrieve gray literature on March 8, 2022. We developed search strings for each database (Appendix). To identify further relevant studies, we checked reference lists of included studies and papers, citing them using the Web of Science database. We also contacted authors for clarification and supplementary information. We applied no restriction in language, publication type, study design, or date in the searches.

We also included unpublished results from a study that included the data reported by McCormick et al. (*11*). The unpublished results (Birch & Davis Associates and Sherikon Inc., US Army Medical Research and Development Command, unpub. data, https://media.tghn.org/medialibrary/2019/03/Responsive_Documents_of_Peter_Horby.pdf.pdf; G.V. Ludwig, pers. comm., 2019 March 4, https://media.tghn.org/medialibrary/2019/03/Dr._Ludwig_memo.pdf) were requested by P.W.H. through the US Freedom of Information Act. We refer to this study as IND 16666, its Food and Drug Administration Investigational New Drug application number.

### Study Selection

We included randomized controlled trials (RCTs), controlled trials, cohort and case–control studies comparing ribavirin treatment with no ribavirin (e.g., supportive treatment) in patients having either or both confirmed and suspected Lassa fever that reported mortality (number of deaths or case-fatality rate). No study reported prespecified secondary outcomes or adverse events, except McCormick et al. (*11*). Therefore, we focused only on mortality in this review.

Two authors independently screened titles and abstracts of retrieved records by using Rayyan (*21*). All records were screened twice, once by the first author (H.C.) and then by 1 of the co-authors (C.E.F., S.D., A.M., and A.P.S.). For records that were potentially eligible, we retrieved and screened the full-text articles, using Excel (Microsoft, https://www.microsoft.com) to record inclusion decisions and manage the workflow. Full-text articles were reviewed independently (H.C. paired with C.E.F. or A.P.S.) to assess the eligibility. We resolved any discrepancies between authors by discussion between the paired assessors. Two authors independently extracted data compiled by 2 authors (H.C. paired with C.E.F. or A.P.S.) by using a prepiloted data extraction form in an Excel spreadsheet.

### Risk for Bias Assessment

Three authors (C.E.F., L.A.M., and H.C.) independently assessed risk for bias for each study by using the ROBINS-I tool (*19*). The tool consists of 7 domains containing a series of signaling questions to judge risk for bias as low, moderate, serious, or critical. For the first domain, we determined bias attributable to confounding or potential confounding factors through a literature review and expert opinion (A.P.S. and P.W.H.). We identified 3 key confounding factors: age, pregnancy status, and indicators of disease severity. For the third domain, bias in classification of interventions, we included assessment of immortal time bias (*22*). We provide support for judgments in individual results (Appendix Table 2).

### Data Analysis and Presentation

As described by Salam et al. (*18*), we used data reported in tables and an appendix within the IND 16666 report to derive aggregated datasets containing the number of deaths according to treatment groups and individual characteristics. On the basis of these datasets, we estimated mortality odds ratios (ORs) comparing ribavirin with no treatment, overall and within subgroups defined by patient characteristics (aminotransferase [AST] level and whether pregnant) in the IND 16666 report. We also extracted results from a logistic regression analysis in the IND 16666 report in which the effect of ribavirin compared with no treatment was adjusted for patient characteristics (age, sex, time to admission, time to treatment, length of stay, and log transformed AST level).

The various reports used different criteria and diagnostic tests to define confirmed Lassa fever cases. Only 1 study, Shaffer et al. (*12*,*15*), provided raw data reporting confirmed Lassa fever according to different case definitions: based on antigen, IgM, and IgG. In this study, we used positive antigen solely as the criteria for the confirmed case because it was consistently reported in the dataset (*15*). We also conducted a sensitivity analyses estimating ORs on the basis of other case definitions.

We estimated overall ORs and, when available, ORs in subgroups defined by timing of treatment (starting <7 and >7 days after disease onset). We did not conduct meta-analyses because most results were rated as at critical overall risk for bias (*19*). We displayed ORs and 95% CIs for the association of ribavirin with no treatment in forest plots by using Stata 15 MP (StataCorp LLC, https://www.stata.com).

## Results

**Figure 1:**
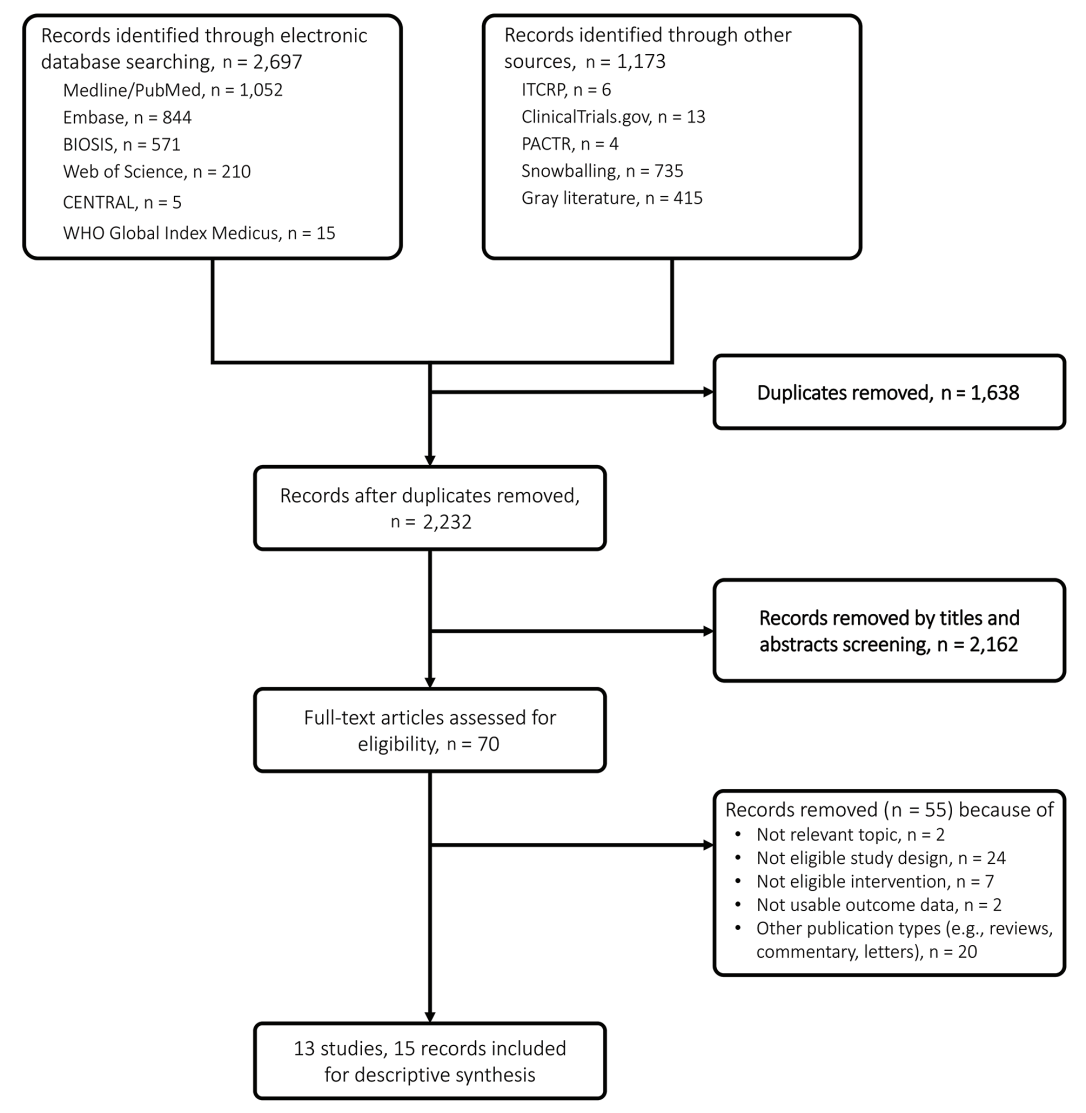
Study selection flowchart for a systematic review of published and unpublished studies for evidence for ribavirin treatment of Lassa fever. ITCRP, World Health Organization International Clinical Trials Registry Platform; PACTR, Pan African Clinical Trial Registry.

We retrieved 2,232 unique records, of which we excluded 2,162 on the basis of titles and abstracts. We retrieved full-text articles for the remaining 70 records for eligibility assessment, after which we excluded 55 further records (Figure 1). One study met the inclusion criteria but was excluded because it reported aggregated outcome data that included unknown treatment status (*23*). Other studies did not report outcome data according to treatment status (*24*–*28*). We contacted the authors for further information but received no responses. We extracted results from 13 eligible studies described in 15 published and unpublished reports and assessed the risk for bias in these results.

### Study Characteristics

We summarized the characteristics of the included studies (Appendix Table 3). All studies were from West Africa (6 from Nigeria and 7 from Sierra Leone) (11,12,14,15,17,29–36; Birch & Davis Associates and Sherikon Inc., US Army Medical Research and Development Command, unpub. data, https://media.tghn.org/medialibrary/2019/03/Responsive_Documents_of_Peter_Horby.pdf.pdf; G.V. Ludwig, pers. comm., 2019 March 4, https://media.tghn.org/medialibrary/2019/03/Dr._Ludwig_memo.pdf; M.-L. Orji et al., unpub. data, https://doi.org/10.20944/preprints202005.0269.v1). McCormick et al. (*11*) and its additional data reported in IND 16666 were described as clinical trials, but we concluded that all studies were observational cohorts, because they did not compare treatment groups that were assigned using randomization. The year of publication ranged from 1986 to 2020. The length of follow up ranged from 1 month to 15 years.

The studies ranged in size from 10 to 1,850 confirmed cases. Most included both child and adult patients, although 2 did not report the characteristics of patients comprehensively (*11*,*34*). Price et al. (*34*) included pregnant women only. Dahmane et al. (*14*) recruited children and women with obstetric conditions. Samuels et al. (*35*) and Orji et al. (M.-L. Orji et al., unpub. data) included children only. Nine of 13 studies were funded by internal or not-for-profit research funders.

Criteria for confirming Lassa fever varied between studies (Appendix Table 4). Real-time PCR was the most common diagnostic test used, followed by virus isolation and Lassa IgM. In IND 16666, the criterion for the no treatment group was either or both bring febrile and having positive Lassa IgG whereas to receive ribavirin participants had to meet 1 of 3 specified diagnostic criteria (Appendix Table 3).

Only 4 studies reported details of ribavirin treatment regimens (*11*,*14*,*38*; Birch & Davis Associates and Sherikon Inc., US Army Medical Research and Development Command, unpub. data, https://media.tghn.org/medialibrary/2019/03/Responsive_Documents_of_Peter_Horby.pdf.pdf; G.V. Ludwig, pers. comm., 2019 March 4, https://media.tghn.org/medialibrary/2019/03/Dr._Ludwig_memo.pdf) (Appendix Table 4). McCormick et al. (*11*) reported 3 ribavirin regimens: 1 oral and 2 intravenous. Dahmane et al. (*14*) reported 1 intravenous ribavirin regimen according to an international guideline. Although 7 ribavirin regimens were reported in IND 16666, the treatment durations and administration routes were not clear. In all studies except Samuels et al. (*35*), detailed information regarding the supportive treatment used was lacking. Three studies reported malaria screening and the use of antimalarial drugs and antibiotics before Lassa fever confirmation (*14*,*34*,*36*).

**Figure 2:**
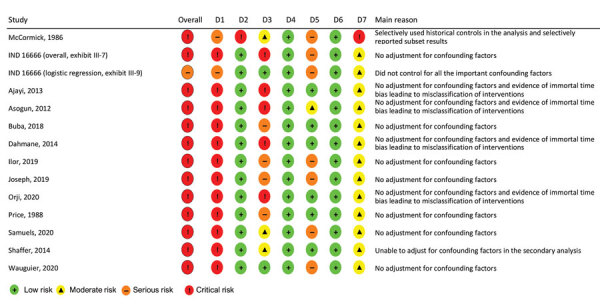
Summary of risk for bias assessment for a systematic review of published and unpublished studies for evidence for ribavirin treatment of Lassa fever. Bias categories: D1, bias due to confounding; D2, bias in selection of participants into the study; D3, bias in classification of interventions; D4, bias due to deviations from intended interventions; D5, bias due to missing data; D6, bias in measurement of outcomes; D7, bias in selection of the reported result. *IND 16666, unpublished study requested by P.W.H. through the US Freedom of Information Act (Birch & Davis Associates and Sherikon Inc., US Army Medical Research and Development Command, unpub. data, https://media.tghn.org/medialibrary/2019/03/Responsive_Documents_of_Peter_Horby.pdf.pdf; G.V. Ludwig, pers. comm., 2019 March 4, https://media.tghn.org/medialibrary/2019/03/Dr._Ludwig_memo.pdf). †M.-L. Orji et al., unpub. data, https://doi.org/10.20944/preprints202005.0269.v1.

We assessed risk for bias in 14 results from 13 studies comparing the effects of ribavirin treatment with no ribavirin treatment on overall mortality outcomes, including 2 results with and without logistic regression adjustment from IND 16666 (Figure 2). The overall risk for bias was rated critical for all results, except for the logistic regression result from IND 16666, which was rated serious.

### Estimated Effects of Ribavirin Treatment on Mortality, Overall and in Subgroups

**Figure 3:**
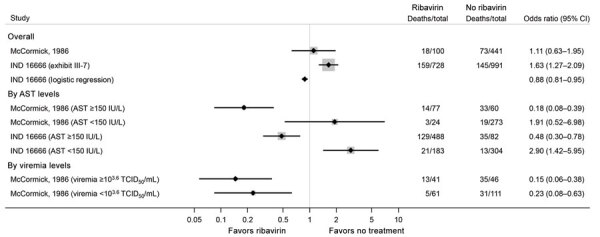
Estimated effects of ribavirin compared with no treatment on mortality outcomes from the McCormick (*11*) and IND 16666 (Birch & Davis Associates and Sherikon Inc., US Army Medical Research and Development Command, unpub. data, https://media.tghn.org/medialibrary/2019/03/Responsive_Documents_of_Peter_Horby.pdf.pdf; G.V. Ludwig, pers. comm., 2019 March 4, https://media.tghn.org/medialibrary/2019/03/Dr._Ludwig_memo.pdf) studies in a systematic review of published and unpublished studies for evidence for ribavirin treatment of Lassa fever. A horizontal line represents the 95% CI of a study result, with each end of the line representing the boundaries. A point estimate of the study result is represented by a black diamond. A gray box gives a representation of the size of a study compared with all studies in the figure.

In the McCormick et al. (*11*) study, for which additional data was reported by IND 16666, ribavirin treatment was associated with higher overall mortality rates in confirmed Lassa fever patients, compared with no ribavirin treatment (Figure 3). However, the IND 16666 study found that, after adjusting for confounding factors using logistic regression, ribavirin was associated with lower overall mortality rates (OR 0.88 [95% CI 0.81–0.95]). We noted that the CI for this logistic regression result appeared too narrow when compared with the unadjusted result derived from the reported numbers of patients and deaths, which was most likely caused by an error in the statistical analysis but could not be checked further.

When results of those studies were stratified by AST levels, ribavirin treatment was associated with lower mortality rates in patients with AST >150 IU/L (OR 0.18 [0.08–0.39] in McCormick et al. [*11*] and OR 0.48 [0.30–0.78] in IND 16666). By contrast, in patients with AST <150 IU/L, ribavirin was associated with higher mortality rates (OR 1.91 [0.52–6.98] in McCormick et al. [*11*] and OR 2.90 [1.42–5.95] in IND 16666 study). In patients with measurable viremia, ribavirin use was associated with lower mortality rates. However, these results should be interpreted with caution because AST or viremia levels were reported to be missing or not measurable in 20%–40% of patients in each study.

**Figure 4:**
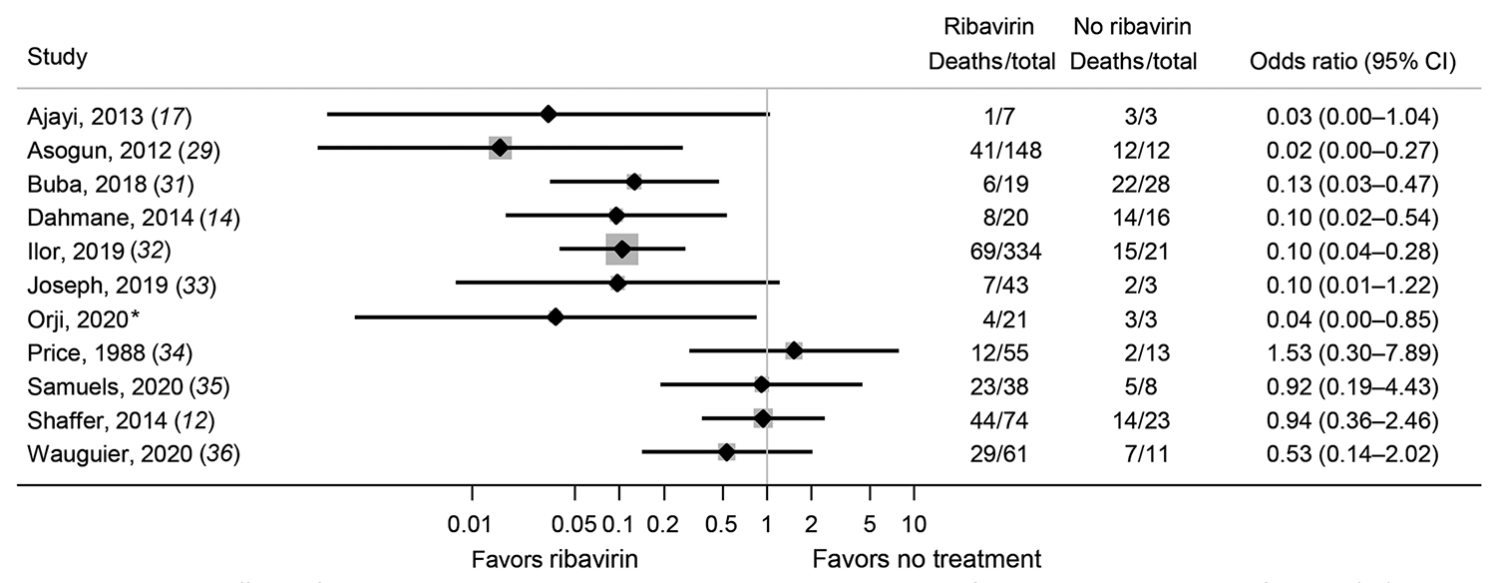
Estimated effects of ribavirin compared with no treatment on mortality outcomes from studies other than McCormick (*11*) and IND 16666 (Birch & Davis Associates and Sherikon Inc., US Army Medical Research and Development Command, unpub. data, https://media.tghn.org/medialibrary/2019/03/Responsive_Documents_of_Peter_Horby.pdf.pdf; G.V. Ludwig, pers. comm., 2019 March 4, https://media.tghn.org/medialibrary/2019/03/Dr._Ludwig_memo.pdf) studies in a systematic review of published and unpublished studies for evidence for ribavirin treatment of Lassa fever. *M.-L. Orji et al., unpub. data, https://doi.org/10.20944/preprints202005.0269.v1. A horizontal line represents the 95% CI of a study result, with each end of the line representing the boundaries. A point estimate of the study result is represented by a black diamond. A gray box gives a representation of the size of a study compared with all studies in the figure.

The other studies mostly found that ribavirin was associated with lower overall mortality rates compared with no ribavirin treatment (Figure 4). However, most of those results were rated as being at critical risk for bias because of lack of adjustment for confounding, immortal time bias, or both (*14*,*17*,*32*; M.-L. Orji et el., unpub. data), which arose because some patients did not receive their intended ribavirin treatment because they died before treatment could be started and were then analyzed in the no treatment group.

**Figure 5:**
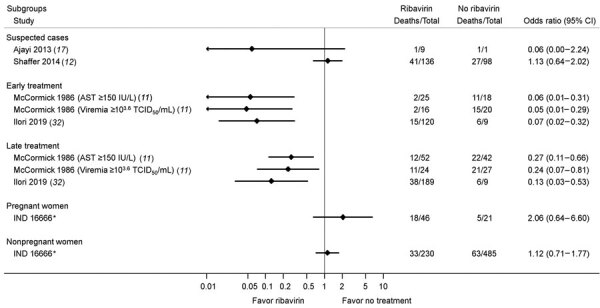
Estimated effects of ribavirin compared with no treatment on mortality outcomes within patient subgroups in a systematic review of published and unpublished studies for evidence for ribavirin treatment of Lassa fever. *IND 16666, unpublished study requested by P.W.H. through the US Freedom of Information Act (Birch & Davis Associates and Sherikon Inc., US Army Medical Research and Development Command, unpub. data, https://media.tghn.org/medialibrary/2019/03/Responsive_Documents_of_Peter_Horby.pdf.pdf; G.V. Ludwig, pers. comm., 2019 March 4, https://media.tghn.org/medialibrary/2019/03/Dr._Ludwig_memo.pdf).

Estimated associations of ribavirin treatment with mortality rates within patient subgroups are reported in the included studies (Figure 5). Many studies included suspected Lassa fever cases, but only 2 studies provided usable data for estimating associations of ribavirin treatment with deaths in suspected cases. Results were discordant; the estimated ORs were 0.06 (95% CI 0.00–2.24) in Ajayi et al. (*17*) and 1.13 (0.64–2.02) in Shaffer et al. (*12*,*15*). We calculated case-fatality rates and ORs from Shaffer et al. (*12*,*15*) on the basis of different case definitions (Appendix Table 5).

Two studies investigated the effects of early versus late ribavirin treatment after disease onset (*11*,*32*). McCormick et al. (*11*) found that in the subgroups AST >150 IU/L and viremia >10^3.6^ 50% median tissue culture infectious dose/mL, the association of ribavirin treatment with a lower mortality rate was more pronounced for treatment within 7 days (early) than at >7 days (late) after disease onset (*11*). Similar results were noted in Ilori et al. (*32*); the ORs were 0.07 (95% CI 0.02–0.32) for early treatment (within 7 days of disease onset) and 0.13 (95% CI 0.03–0.53)] for late treatment (>7 days after disease onset).

Only 1 study provided a result of subgroup analysis to compare pregnant women with nonpregnant women. The IND 16666 study reported separate results for pregnant women (OR 2.06 [95% CI 0.64–6.60]) and nonpregnant women (OR 1.12 [95% CI 0.71–1.77]).

## Discussion

This systematic review summarizes associations of ribavirin treatment, compared with no ribavirin treatment, with overall mortality outcomes in confirmed Lassa fever, using both published and unpublished study results. Although ribavirin treatment was generally associated with lower mortality rates, almost all results were rated as being at critical risk for bias. In the single adjusted result from the IND 16666 study, ribavirin was associated with modestly lower mortality rates. However, that result was assessed as being at serious risk for bias, and the CI appeared too narrow compared with the CI derived from the numbers of patients and deaths. Although ribavirin was reported to be associated with lower mortality rates in certain subgroups, including patients with AST >150 IU/L and measurable viremia, missing data and the post-hoc nature of the analyses limit the credibility of these findings. By contrast, ribavirin was reported to be associated with higher mortality rates than ribavirin treatment in other subgroups, such as patients with AST <150 IU/L. In summary, it is uncertain based on the available literature whether ribavirin reduces mortality rates in Lassa fever patients.

For decades, ribavirin has been used to treat Lassa fever, supported in particular by the results of the McCormick study (*11*). However, treatment guidelines generally do not highlight the weakness of the primary evidence, nor do they distinguish patient subgroups (e.g., patients with AST <150 IU/L) where benefit has not been demonstrated and, in fact, there may be hazard from using ribavirin (*37*,*38*). Because ribavirin causes adverse events and is expensive (up to 5,000€/patient) (*14*,*37*), it is important to justify its use in treating Lassa fever, especially in low- and middle-income countries where healthcare resources are limited. Although such uncertainty exists in the efficacy and safety of ribavirin, we believe that it is important to firmly establish evidence of efficacy and safety by conducting randomized controlled clinical trials. For example, WHO has identified the need for a multicenter phase 2b/3 RCT with 2 possible designs: a 4-arm factorial design with ribavirin and best supportive care and a 3-arm RCT with ribavirin, best supportive care, and another drug (*39*). In line with this approach, a combination of ribavirin and favipiravir treatment has been proposed by Raabe et al. (*40*)

Our findings agree with those of a previous systematic review (*41*). Both reviews identified a need to reevaluate the safety and efficacy of ribavirin for Lassa fever. In comparison with the prior review (which included studies published up to March 2019), our study included 6 additional studies, presented more detailed results (including secondary analyses), and provided a more detailed evaluation of the potential biases in study results.

Our review was conducted using state-of-the-art systematic review methodology. We conducted comprehensive literature searches, including a range of electronic databases and gray literature, without date, language, or study design restrictions. We used the ROBINS-I tool (*19*) for risk for bias assessments; this tool is the most comprehensive and widely used tool for assessing risk for bias in the results of nonrandomized studies of interventions. Our review incorporated recent changes to ROBINS-I that address immortal time bias; evidence of such bias was identified in several of the included studies.

We conducted secondary analyses of the related McCormick (*11*) and IND 16666 studies. To estimate overall associations of ribavirin treatment with mortality outcomes, we grouped different ribavirin treatment regimens and routes of administration. Treatment efficacies might differ between these regimens, but it was challenging to distinguish the ribavirin regimens used in these studies because their details were not fully described. There may have been differences in the care given to the no ribavirin treatment groups across studies; such care could be no medical support, minimal medical support, or supportive treatment, and the type of care is likely to have varied over time, by country and by setting. We did not perform subgroup analyses, investigating the implications of different criteria used to define Lassa fever, because except for Shaffer et al. (*12*,*15*), no studies provided data that could be used for subgroup analyses. We only identified studies conducted in Nigeria and Sierra Leone, but Lassa fever is endemic in several other countries in West Africa.

These findings have important implications for both clinical practice and research. The serious limitations of the available evidence means that although the studies we reviewed suggest an association of ribavirin treatment for Lassa fever with decreased mortality rates, this conclusion must be viewed with limited confidence. Evidence from high-quality randomized trials is urgently required, and clinical and research communities should work collaboratively to address and overcome ethics and resource issues to fund and conduct such trials in West Africa.

Appendix Additional information about evidence for ribavirin treatment of Lassa fever in a systematic review of published and unpublished studies.
