# Supplementary material for: Lack of Evidence for Ribavirin Treatment of Lassa Fever in Systematic Review of Published and Unpublished Studies
Source: Emerg Infect Dis. 2022 Aug;28(8):1559–68. doi: 10.3201/eid2808.211787 (PMC9328902; doi:10.3201/eid2808.211787)
Supplement: Appendix — Additional information about evidence for ribavirin treatment of Lassa fever in a systematic review of published and unpublished studies. [file 21-1787-Techapp-s1.pdf]

Article DOI: https://doi.org/10.3201/eid2808.211787

Lack of Evidence for Ribavirin Treatment of Lassa Fever in a Systematic Review of Published and Unpublished Studies

Appendix

Search Strategies

Ovid MEDLINE(R) ALL <1946 to March 8 2022>

1. Lassa Fever/
2. Lassa virus/
3. (lassa adj3 (infect* or fever or virus* or viral or arenavir* or outbreak?)).ti,ab,kf.
4. lassa.ti,ot.
5. (LASV or Lassa mammarenavirus).mp.
6. or/1-5
7. Ribavirin/
8. (ribavirin* or tribavirin* or viramidin* or ribamidin* or Copegus or Ibavyr or Moderiba or Rebetol).mp.
9. Disease Management/ or Drug Evaluation/ or Infection Control/ or Treatment Outcome/
10. (drug therapy or prevention & control or therapy).fs.
11. (death? or disease outbreak? or mortalit* or survival or survivor?).kf,hw.
12. case fatalit*.mp.
13. or/7-12
14. 6 and 13

PubMed NOT MEDLINE (All years to 8 Mar 2022)

#1 Search ("LASSA FEVER"[Mesh:NoExp]) OR "LASSA VIRUS"[Mesh:NoExp]

#2 “lassa fever” OR “lassa hemorrhagic fever” OR “lassa haemorrhagic fever” OR “lassa virus” or LASV OR “Lassa mammarenavirus”

#3 (#1 OR #2)

#4 pubmednotmedline[sb]

#5 publisher[sb] NOT pubstatusnihms NOT pubstatuspmcsd NOT pmcbook

#6 (#4 OR #5)

#7 (#3 AND #6)

Ovid Embase <1980 to 2022 March 08>

1 Lassa fever/

2 Lassa virus/

3 (lassa adj3 (infect* or fever or virus* or viral or arenavir* or outbreak?)).ti,ab,kw.

4 lassa.ti,ot.

5 (LASV or Lassa mammarenavirus).ti,ab,kw.

6 Arenavirus Infection/

7 old world arenavirus/ or mammarenavirus/

8 or/1-7

9 ribavirin/

10 (ribavirin* or tribavirin* or viramidin* or ribamidin* or Copegus or Ibavyr or Moderiba or Rebetol).mp.

11 drug therapy.dy,fs,kw,ox,xw.

12 (drug adj (administration or comparison or efficacy or therapy)).hw.

13 antivirus agent/

14 INFECTION CONTROL/ or TREATMENT OUTCOME/

15 or/9-14

16 8 and 15

Web of Science (All years to 8 Mar 2022)

• Science Citation Index Expanded (SCI-EXPANDED) --1900-present

• Conference Proceedings Citation Index- Science (CPCI-S) --1990-present

#1 TITLE: (lassa) OR TOPIC: ((lassa SAME (infect* or fever or virus* or viral or arenavir* or outbreak*))) OR TOPIC: (LASV)

#2 (ribavirin* or tribavirin* or viramidin* or ribamidin* or Copegus or Ibavyr or Moderiba or Rebetol)

#3 (#1 and #2)

BIOSIS Citation Index (BCI) (2020 to 8 March 2022)

#1 TOPIC: ((lassa SAME (infect* or fever or virus* or viral or arenavir* or outbreak*))) AND TOPIC: (ribavirin* or tribavirin* or viramidin* or ribamidin* or Copegus or Ibavyr or Moderiba or Rebetol)

#2 TITLE: (lassa or LASV)

#3 TAXONOMIC DATA: (Hominidae [86215])

#4 (#2 AND #3)

#5 (#1 OR #4)

Central Register of Controlled Trials (CENTRAL) on the Cochrane Library, Issue 3 of 12, 2022

(lassa or LASV) AND (ribavirin* OR tribavirin* OR viramidin* OR ribamidin* OR Copegus OR Ibavyr OR Moderiba OR Rebetol) [all fields]

WHO Global Index Medicus

S1 (lassa or LASV)

S2 (ribavirin* OR tribavirin* OR viramidin* OR ribamidin* OR Copegus OR Ibavyr OR Moderiba OR Rebetol)

S3 (S1 or S2)

WHO International Clinical Trials Registry Platform (ICTRP)

(lassa and ribavirin* or lassa and tribavirin* or lassa and viramidin* or lassa and ribamidin* or lassa and Copegus or lassa and ibavyr or lassa and moderiba or lassa and rebetol or LASV and ribavirin* or LASV and tribavirin* or LASV and viramidin* or LASV and ribamidin* or LASV and copegus or LASV and ibavyr or LASV and moderiba or LASV and rebetol)

ClinicalTrials.gov

(lassa OR LASV)

Pan African Clinical Trials Registry (PACTR) (https://pactr.samrc.ac.za)

S1 Lassa

S2 LASV

S3 Ribavirin

S4 Arenavirus

OR/S1-S4

Note: Additional scoping searches were conducted on *LILACS*, but no relevant records were retrieved.

Summary of Judgments on Risk of Bias Assessment

Protocol stage

- Participants: Patients, regardless of age, with confirmed (e.g. PCR, Lassa Ag + or IgM positive) or suspected Lassa fever
- Experimental intervention: Any treatment regimen or administration routes (e.g. intravenous or oral) of ribavirin for treating or preventing Lassa fever
- Comparator: Placebo, supportive care, no treatment or other intervention. Supportive care includes any supportive interventions for treating or relieving symptoms of Lassa fever, such as respiratory distress, hemorrhaging and organ failure.
- Outcome: Mortality

List of the confounding factors relevant to all or most studies

- Age
- Pregnancy status
- Biomarkers/signs/symptoms of disease severity

Aim for each study

- To assess the effect of assignment to intervention

Secondary Analyses

The full data set includes 1740 observations. The details of eligibility criteria for the data set can be found in Shafer et al (*1*,*2*). In this review, we were concerned with effect of ribavirin compared with no treatment, on survival. Thus, only 373 of 1740 patients were eligible (because they had both treatment status and survival recorded). Among these 373 patients, all those with admission status 'Not admitted' died (n = 42), providing no efficacy comparison, so we excluded them. This left 331 patients (suspected and confirmed cases) with treatment status, survival outcome, and 'admitted' status for the secondary analysis.

Shaffer et al. reported three types of serostatus according to antigen (Ag), immunoglobulin M (IgM), and immunoglobulin G (IgG) ELISA tests for determining Lassa fever. In our main results we use positive Ag (Ag+) as the criterion for Lassa fever confirmation, after discussion with clinical experts.

In sensitivity analyses, we explored the following criteria for confirmed Lassa fever cases. There were discrepancies in IgM serostatus between serostatus 1 and serostatus 2. Thus, we presented two results for IgM serostatus:

1. IgM+ only (IgM+ in serostatus 1 or 2)
2. IgM+ only (IgM+ in serostatus 1 and 2)
3. IgG+ only
4. Ag+ or IgM+ (IgM+ in serostatus 1 or 2)
5. Ag+ or IgM+ (IgM+ in serostatus 1 and 2)
6. Ag+ or IgG+

7. IgM+ or IgG+ positive (IgM+ in serostatus 1 or 2)

8. IgM+ or IgG+ positive (IgM+ in serostatus 1 and 2)

9. Ag+, IgM+ or IgG+

Next, we explored suspected cases in the following criteria:

1. Ag- only

2. IgM- only (IgM- in serostatus 1 and 2)

3. IgM- only (IgM- in serostatus 1 or 2)

4. IgG- only

5. Ag- or IgM- (IgM- in serostatus 1 and 2)

6. Ag- or IgM- (IgM- in serostatus 1 or 2)

7. Ag- or IgG-

8. IgM- or IgG- positive (IgM+ in serostatus 1 and 2)

9. IgM- or IgG- positive (IgM+ in serostatus 1 or 2)

10. Ag-, IgM- or IgG-

Last, we conducted an all-case analysis (regardless of ELISA test results) (Appendix Table 5).

References

1. Shaffer JG, Grant DS, Schieffelin JS, Boisen ML, Goba A, Hartnett JN, et al.; Viral Hemorrhagic Fever Consortium. Lassa fever in post-conflict sierra leone. PLoS Negl Trop Dis. 2014;8:e2748. PubMed https://doi.org/10.1371/journal.pntd.0002748

2. Shaffer JG, Schieffelin JS, Grant DS, Goba A, Momoh M, Kanneh L, et al.; Viral Hemorrhagic Fever Consortium. Data set on Lassa fever in post-conflict Sierra Leone. Data Brief. 2019;23:103673. PubMed https://doi.org/10.1016/j.dib.2019.01.021

3. Moher D, Liberati A, Tetzlaff J, Altman DG; PRISMA Group. Preferred reporting items for systematic reviews and meta-analyses: the PRISMA statement. PLoS Med. 2009;6:e1000097. PubMed https://doi.org/10.1371/journal.pmed.1000097

4. Ajayi NA, Nwigwe CG, Azuogu BN, Onyire BN, Nwonwu EU, Ogbonnaya LU, et al. Containing a Lassa fever epidemic in a resource-limited setting: outbreak description and lessons learned from Abakaliki, Nigeria (January-March 2012). Int J Infect Dis. 2013;17:e1011–6. PubMed https://doi.org/10.1016/j.ijid.2013.05.015

5. Asogun DA, Adomeh DI, Ehimuan J, Odia I, Hass M, Gabriel M, et al. Molecular diagnostics for lassa fever at Irrua specialist teaching hospital, Nigeria: lessons learnt from two years of laboratory operation. PLoS Negl Trop Dis. 2012;6:e1839. PubMed https://doi.org/10.1371/journal.pntd.0001839

6. Buba MI, Dalhat MM, Nguku PM, Waziri N, Mohammad JO, Bomoi IM, et al. Mortality among confirmed Lassa fever cases during the 2015–2016 outbreak in Nigeria. Am J Public Health. 2018;108:262–4. PubMed https://doi.org/10.2105/AJPH.2017.304186

7. Dahmane A, van Griensven J, Van Herp M, Van den Bergh R, Nzomukunda Y, Prior J, et al. Constraints in the diagnosis and treatment of Lassa Fever and the effect on mortality in hospitalized children and women with obstetric conditions in a rural district hospital in Sierra Leone. Trans R Soc Trop Med Hyg. 2014;108:126–32. PubMed https://doi.org/10.1093/trstmh/tru009

8. Bouree P. Les parasitoses intestinales sont encore fréquentes. Med Sante Trop. 2015;25:130. https://doi.org/10.1684/mst.2015.0459

9. Ilori EA, Furuse Y, Ipadeola OB, Dan-Nwafor CC, Abubakar A, Womi-Eteng OE, et al.; Nigeria Lassa Fever National Response Team. Epidemiologic and clinical features of Lassa fever outbreak in Nigeria, January 1–May 6, 2018. Emerg Infect Dis. 2019;25:1066–74. PubMed https://doi.org/10.3201/eid2506.181035

10. Joseph A, Robinson O, Justus E, Matthew N, Chukwuemeka U. Clinical profile of Lassa fever patients in Abakaliki, south-eastern Nigeria, January–March 2018. Ann Med Health Sci Res. 2019;9:598–602.

11. McCormick JB, King IJ, Webb PA, Scribner CL, Craven RB, Johnson KM, et al. Lassa fever. Effective therapy with ribavirin. N Engl J Med. 1986;314:20–6. PubMed https://doi.org/10.1056/NEJM198601023140104

12. Price ME, Fisher-Hoch SP, Craven RB, McCormick JB. A prospective study of maternal and fetal outcome in acute Lassa fever infection during pregnancy. BMJ. 1988;297:584–7. PubMed https://doi.org/10.1136/bmj.297.6648.584

13. Samuels RJ, Moon TD, Starnes JR, Alhasan F, Gbakie M, Goba A, et al. Lassa fever among children in Eastern Province, Sierra Leone: a 7-year retrospective analysis (2012–2018). Am J Trop Med Hyg. 2020;104:585–92. PubMed https://doi.org/10.4269/ajtmh.20-0773

14. Wauquier N, Couffignal C, Manchon P, Smith E, Lungay V, Coomber M, et al. High heart rate at admission as a predictive factor of mortality in hospitalized patients with Lassa fever: An observational cohort study in Sierra Leone. J Infect. 2020;80:671–93. PubMed https://doi.org/10.1016/j.jinf.2020.01.021

15. World Health Organization. Recommendations for management of common childhood conditions: evidence for technical update of pocket book recommendations: newborn conditions, dysentery, pneumonia, oxygen use and delivery, common causes of fever, severe acute malnutrition and supportive care. 2012 [cited 2022 Mar 22]. https://apps.who.int/iris/handle/10665/44774

16. World Health Organization. Paediatric emergency triage, assessment and treatment (ETAT), care of critically ill children. 2016 [cited 2022 Mar 22]. https://www.who.int/publications/i/item/9789241510219

Appendix Table 1. PRISMA 2009 Checklist (*3*)

Section/topic	#	Checklist item	Reported on page #
Title			
Title	1	Identify the report as a systematic review, meta-analysis, or both.	1
Abstract			
Structured summary	2	Provide a structured summary including, as applicable: background; objectives; data sources; study eligibility criteria, participants, and interventions; study appraisal and synthesis methods; results; limitations; conclusions and implications of key findings; systematic review registration number.	2
Introduction			
Rationale	3	Describe the rationale for the review in the context of what is already known.	2-3
Objectives	4	Provide an explicit statement of questions being addressed with reference to participants, interventions, comparisons, outcomes, and study design (PICOS).	2-3
Methods			
Protocol and registration	5	Indicate if a review protocol exists, if and where it can be accessed (e.g., Web address), and, if available, provide registration information including registration number.	3
Eligibility criteria	6	Specify study characteristics (e.g., PICOS, length of follow-up) and report characteristics (e.g., years considered, language, publication status) used as criteria for eligibility, giving rationale.	4
Information sources	7	Describe all information sources (e.g., databases with dates of coverage, contact with study authors to identify additional studies) in the search and date last searched.	3-4
Search	8	Present full electronic search strategy for at least one database, including any limits used, such that it could be repeated.	Appendix
Study selection	9	State the process for selecting studies (i.e., screening, eligibility, included in systematic review, and, if applicable, included in the meta-analysis).	4-5
Data collection process	10	Describe method of data extraction from reports (e.g., piloted forms, independently, in duplicate) and any processes for obtaining and confirming data from investigators.	4-5
Data items	11	List and define all variables for which data were sought (e.g., PICOS, funding sources) and any assumptions and simplifications made.	4-5
Risk of bias in individual studies	12	Describe methods used for assessing risk of bias of individual studies (including specification of whether this was done at the study or outcome level), and how this information is to be used in any data synthesis.	5
Summary measures	13	State the principal summary measures (e.g., risk ratio, difference in means).	5-6
Synthesis of results	14	Describe the methods of handling data and combining results of studies, if done, including measures of consistency (e.g., I^2) for each meta-analysis.	5-6
Risk of bias across studies	15	Specify any assessment of risk of bias that may affect the cumulative evidence (e.g., publication bias, selective reporting within studies).	6
Additional analyses	16	Describe methods of additional analyses (e.g., sensitivity or subgroup analyses, meta-regression), if done, indicating which were pre-specified.	6
Results			
Study selection	17	Give numbers of studies screened, assessed for eligibility, and included in the review, with reasons for exclusions at each stage, ideally with a flow diagram.	Fig 1
Study characteristics	18	For each study, present characteristics for which data were extracted (e.g., study size, PICOS, follow-up period) and provide the citations.	Tab 1
Risk of bias within studies	19	Present data on risk of bias of each study and, if available, any outcome level assessment (see item 12).	Fig 2
Results of individual studies	20	For all outcomes considered (benefits or harms), present, for each study: (a) simple summary data for each intervention group (b) effect estimates and confidence intervals, ideally with a forest plot.	Fig 3, 4
Synthesis of results	21	Present results of each meta-analysis done, including confidence intervals and measures of consistency.	Fig 3, 4
Risk of bias across studies	22	Present results of any assessment of risk of bias across studies (see Item 15).	Tab 1, Tab 2
Additional analysis	23	Give results of additional analyses, if done (e.g., sensitivity or subgroup analyses, meta-regression [see Item 16]).	Fig 4-5
Discussion			
Summary of evidence	24	Summarize the main findings including the strength of evidence for each main outcome; consider their relevance to key groups (e.g., healthcare providers, users, and policy makers).	9
Limitations	25	Discuss limitations at study and outcome level (e.g., risk of bias), and at review-level (e.g., incomplete retrieval of identified research, reporting bias).	10-11
Conclusions	26	Provide a general interpretation of the results in the context of other evidence, and implications for future research.	11-12
Funding			
Funding	27	Describe sources of funding for the systematic review and other support (e.g., supply of data); role of funders for the systematic review.	12

Appendix Table 2. Summary of risk of bias assessment

	Study													
Domain	McCormick 1986	IND 16666 (Overall)	IND 16666 (Logistic regression)	Ajayi 2013	Asogun 2012	Buba 2018	Dahmane 2014	Ilori 2019	Joseph 2019	Price 1988	Shaffer 2014	Wauguier 2020	Orji 2020	Samuels 2020
Overall bias	Critical	Critical	Serious	Critical	Critical	Critical	Critical	Critical	Critical	Critical	Critical	Critical	Critical	Critical
Bias due to confounding	Serious<br>Authors explored effects of some confounding factors on the outcome but did not control for all the important confounding domains. (Q1.4)	Critical<br>No adjustments for confounding factors.	Serious<br>Authors explored effects of some confounding factors on the outcome but did not control for all the important confounding domains. (Q1.4)	Critical<br>No adjustments for confounding factors.	Critical<br>No adjustments for confounding factors.	Critical<br>No adjustments for confounding factors.	Critical<br>No adjustments for confounding factors.	Critical<br>No adjustments for confounding factors.	Critical<br>No adjustments for confounding factors.	Critical<br>No adjustments for confounding factors.	Critical<br>Unable to adjust for confounding factors in the secondary analysis.	Critical<br>No adjustments for confounding factors on ribavirin and controls.	Critical<br>No adjustments for confounding factors.	Critical<br>No adjustments for confounding factors.
Bias in selection of participants into the study	Critical<br>The authors used historical controls in the analysis without providing further information nor justification. (Q 2.1 & 2.4)	Low<br>Participants were not selected nor analyzed based participant characteristics observed after the start of intervention. Start of follow-up and start of intervention coincide for most participants.	Low<br>Participants were not selected nor analyzed based participant characteristics observed after the start of intervention. Start of follow-up and start of intervention coincide for most participants.	Low<br>Participants were not selected nor analyzed based participant characteristics observed after the start of intervention. Start of follow-up and start of intervention coincide for most participants.	Low<br>Participants were not selected nor analyzed based participant characteristics observed after the start of intervention. Start of follow-up and start of intervention coincide for most participants.	Low<br>Participants were not selected nor analyzed based participant characteristics observed after the start of intervention. Start of follow-up and start of intervention coincide for most participants.	Low<br>Participants were not selected nor analyzed based participant characteristics observed after the start of intervention. Start of follow-up and start of intervention coincide for most participants.	Low<br>Participants were not selected nor analyzed based participant characteristics observed after the start of intervention. Start of follow-up and start of intervention coincide for most participants.	Low<br>Participants were not selected nor analyzed based participant characteristics observed after the start of intervention. Start of follow-up and start of intervention coincide for most participants.	Low<br>Participants were not selected nor analyzed based participant characteristics observed after the start of intervention. Start of follow-up and start of intervention coincide for most participants.	Low<br>Participants were not selected nor analyzed based participant characteristics observed after the start of intervention. Start of follow-up and start of intervention coincide for most participants.	Low<br>Participants were not selected nor analyzed based participant characteristics observed after the start of intervention. Start of follow-up and start of intervention coincide for most participants.	Low<br>Participants were not selected nor analyzed based participant characteristics observed after the start of intervention. Start of follow-up and start of intervention coincide for most participants.	Low<br>Participants were not selected nor analyzed based participant characteristics observed after the start of intervention. Start of follow-up and start of intervention coincide for most participants.
Bias in classification of interventions	Moderate<br>The authors combined certain groups in the later analysis, "*We observed a case-fatality rate of 29 percent (9 of 31) in patients*	Critical<br>"*The treated group were more severely ill and, thus, they would be at a disadvantage in terms of survival.*" – suggesting	Low<br>Immortal time bias in classification of interventions was adjusted by logistic regression.	Critical<br>"*Four of the patients who died during the outbreak did not receive ribavirin therapy. The index case was not*	Critical<br>"*…23% of Lassa fever patients with fatal outcome did not receive ribavirin because they died the day of*	Serious<br>*"We defined ribavirin commencement as early if it was started within 7 days of symptom onset and as delayed if it was not."* –	Critical<br>"*Of 16 patients who did not receive ribavirin, 14 (87%) died before ribavirin treatment could be*	Serious<br>*"Patients in severe conditions might have not received ribavirin because they died before reaching healthcare*	Serious<br>"Although there is an improved case detection and access to ribavirin, some patients still presented late to the hospital.	Serious<br>"*Women less than 20 weeks pregnant suspected of having Lassa fever were admitted to hospital and treated on the general*	Moderate<br>Classification of interventions was derived from clinical records (administration of ribavirin therapy observed	Low<br>No evidence of bias in classification of interventions nor immortal time bias.	Critical<br>"A total of 12.5% of the children that tested positive to Lassa virus PCR and were unable to receive ribavirin	Moderate<br>Classification of interventions was derived from clinical records (administration of ribavirin therapy observed

	Study													
Domain	McCormick 1986	IND 16666 (Overall)	IND 16666 (Logistic regression)	Ajayi 2013	Asogun 2012	Buba 2018	Dahmane 2014	Ilori 2019	Joseph 2019	Price 1988	Shaffer 2014	Wauguier 2020	Orji 2020	Samuels 2020
	treated with 1 unit of Lassa-convalescent plasma; this rate did not differ significantly from the rate in patients treated with 2 units of plasma (36 percent, 8 of 22). Hence, we combined both these patient groups for analysis as the plasma-treated group (53 patients)." (Q 3.2)	immortal time bias in classification of interventions (Q 3.3)		*treated because the confirmatory diagnosis did not return until her death. The other three patients died within a few hours of presentation.*" – suggesting immortal time bias on classification of interventions (Q 3.3)	*presentation or the next day.*" – suggesting immortal time bias on classification of interventions (Q 3.3)	suggesting a possibility of immortal time bias (Q 3.3)	*commenced."* – suggesting immortal time bias in classification of interventions (Q 3.3)	*facilities where treatment was available…"* – authors recognized the situation in the discussion, implying potential immortal time bias in classification of interventions (Q 3.3)	Majority of the fatalities occurred among health workers." – suggesting immortal time bias in classification of interventions (Q 3.3)	*female ward. If the clinical diagnosis was strongly suspected or had been confirmed by serologic testing, the patient was transferred to an isolation room."* – suggesting some aspects of treatment status depending on pregnancy (Q 3.3)	during hospitalization).		medication died." – authors recognized the situation in the discussion, implying potential immortal time bias in classification of interventions (Q 3.3)	during hospitalization). And "It is not clear why only 66% (38/57) of our cohort with LF antigen received ribavirin" – suggesting a chance of intervention given by patient's status.
Bias due to deviations from intended interventions	Low<br>No or few deviations from the intended intervention.	Low<br>No or little deviations from the intended intervention due to a retrospective study design.	Low<br>No or little deviations from the intended intervention due to a retrospective study design.	Low<br>None or little deviations from the intended intervention due to retrospective study design.	Low<br>None or little deviations from the intended intervention due to retrospective study design.	Low<br>None or little deviations from the intended intervention due to retrospective study design.	Low<br>No or little deviations from the intended intervention due to a retrospective study design.	Low<br>No or little deviations from the intended intervention due to a retrospective study design.	Low<br>No or little deviations from the intended intervention due to a retrospective study design.	Low<br>No or few deviations from the intended intervention.	Low<br>No or few deviations from the intended intervention.	Low<br>No or few deviations from the intended intervention.	Low<br>No or few deviations from the intended intervention.	Low<br>No or few deviations from the intended intervention.
Bias due to missing data	Serious<br>Complete data were not available for all participants due to the use of historical controls.	Serious<br>There were only 1795/2154 (83.3%) cases reported with survivorship in Table Exhibit III-7. (Q 5.1)	Serious<br>There were missing data on survivorship and SGOT levels.	Low<br>No evidence of missing data was found.	Moderate<br>There were missing data (161/183) and no reasons were given. However, the proportion of missing in both groups is similar, 21/169 (ribavirin) vs	Low<br>No evidence of missing data was found.	Low<br>No evidence of missing data was found.	Serious<br>There were only 355/414 (85.7%) cases reported with treatment status. (Q 5.1)	Serious<br>62 confirmed cases but only 46 cases with unknown treatment status (Q 5.1)	Low<br>No evidence of missing data was found.	Low<br>No evidence of missing data was found.	Serious<br>79 confirmed cases but only 72 cases with unknown treatment status.	Low<br>No evidence of missing data was found.	Serious<br>Intervention status was missing for 11/57 patients (19%) who were excluded (6 in the 'survived' group (21%) and 13 (36%) in the 'died' group).

	Study													
Domain	McCormick 1986	IND 16666 (Overall)	IND 16666 (Logistic regression)	Ajayi 2013	Asogun 2012	Buba 2018	Dahmane 2014	Ilori 2019	Joseph 2019	Price 1988	Shaffer 2014	Wauguier 2020	Orji 2020	Samuels 2020
					2/14 (no ribavirin). (Q 5.1)									
Bias in measurement of outcomes	Low<br>The outcome measure, death, was unlikely to be influenced by knowledge of the intervention or present systematic errors.	Low<br>The outcome measure, death, was unlikely to be influenced by knowledge of the intervention or present systematic errors.	Low<br>The outcome measure, death, was unlikely to be influenced by knowledge of the intervention or present systematic errors.	Low<br>The outcome measure, death, was unlikely to be influenced by knowledge of the intervention or present systematic errors.	Low<br>The outcome measure, death, was unlikely to be influenced by knowledge of the intervention or present systematic errors.	Low<br>The outcome measure, death, was unlikely to be influenced by knowledge of the intervention or present systematic errors.	Low<br>The outcome measure, death, was unlikely to be influenced by knowledge of the intervention or present systematic errors.	Low<br>The outcome measure, death, was unlikely to be influenced by knowledge of the intervention or present systematic errors.	Low<br>The outcome measure, death, was unlikely to be influenced by knowledge of the intervention or present systematic errors.	Low<br>The outcome measure, death, was unlikely to be influenced by knowledge of the intervention or present systematic errors.	Low<br>The outcome measure, death, was unlikely to be influenced by knowledge of the intervention or present systematic errors.	Low<br>The outcome measure, death, was unlikely to be influenced by knowledge of the intervention or present systematic errors.	Low<br>The outcome measure, death, was unlikely to be influenced by knowledge of the intervention or present systematic errors.	Low<br>The outcome measure, death, was unlikely to be influenced by knowledge of the intervention or present systematic errors.
Bias in selection of the reported result	Critical<br>Lack of protocol. The authors did post-hoc decisions on their analysis and emphasized on subgroup results.	Moderate<br>Lack of protocol but no evidence of bias in selection of the reported result	Moderate<br>Lack of protocol but no evidence of bias in selection of the reported result	Moderate<br>Lack of protocol but no evidence of bias in selection of the reported result	Moderate<br>Lack of protocol but no evidence of bias in selection of the reported result	Moderate<br>Lack of protocol but no evidence of bias in selection of the reported result	Moderate<br>Lack of protocol but no evidence of bias in selection of the reported result	Moderate<br>Lack of protocol but no evidence of bias in selection of the reported result	Moderate<br>Lack of protocol but no evidence of bias in selection of the reported result	Moderate<br>Lack of protocol but no evidence of bias in selection of the reported result	Moderate<br>Lack of protocol but no evidence of bias in selection of the reported result	Moderate<br>Lack of protocol but no evidence of bias in selection of the reported result	Moderate<br>Lack of protocol but no evidence of bias in selection of the reported result	Moderate<br>Lack of protocol but no evidence of bias in selection of the reported result

Appendix Table 3. Characteristics of studies

Study	Country	Study period	Design	No. of patients (% male)	Population; Age (year)	Criteria for confirming Lassa fever cases	Funding
Ajayi 2013 (*4*)	Nigeria	Jan 2012 - Mar 2012	Cohort	10* (70%)	Children and adults; Median: 36 (range 12-47)	Positive Lassa IgM antibody, PCR, or virus isolation	German Research Foundation and WHO
Asogun 2012 (*5*)	Nigeria	Jan 2009 - Dec 2010	Cohort	198* (51.3%)	Adults; Median: 32 (IQR 23-46)	RT-PCR	Volkswagen Foundation, German Research Foundation, European Community and Harvard University
Buba 2018 (*6*)	Nigeria	Oct 2015 - Feb 2016	Cohort	47 (63.8%)	Children and Adults; Mean: 31.4 (SD 18.4)	RT-PCR or ELISA	NR
Dahmane 2014 (*7*,*8*)	Sierra Leone	Apr 2011 - Feb 2012	Cohort	36* (55.6%)	Children and women with obstetric conditions; Age<15 yrs: 80%	Positive Lassa virus Ag or Lassa IgM antibody	An anonymous donor, Department for International Development, UK and Medecins Sans Frontieres
Ilori 2019 (*9*)	Nigeria	Jan – May 2018	Cohort	423 (62.1%)	Children and adults; Age 0-20 yrs: 26.2%	Positive IgM, RT-PCR, or virus isolation	NR
IND 16666†	Sierra Leone	1977 – 1991	Cohort	1850* (45.6%)	Children and adults; Age<15 yrs: 7.1%	Confirmed by the CDC; or an IFA reading of 30 or more; or had a positive viremia, IgG, IgM; or had a positive liver touch prep (21 p16)	Ministry of Health of Sierra Leone and Centers for Disease Control (CDC) and the U.S. Army Medical Research and Development Command
Joseph 2019 (*10*)	Nigeria	March 2018	Cohort	62 (36.2%)	Children and adults; Age 0-19 yrs: 18.8%	RT-PCR	NR
McCormick 1986 (*11*)	Sierra Leone	Feb 1977 – Jan 1979	Controlled study	596 (NR)	Children and adults; NR	Virus isolation from serum or other body fluids/organs, IFA titers <1:4 to ≥1:16, or Lassa antibody titer ≥1:256 and Lassa IgM antibody titer ≥1:16	Ministry of Health of Sierra Leone and Centers for Disease Control (CDC)
Orji 2020‡	Nigeria	Jan 2019 – Jan 2020	Cohort	24* (37.5%)	Children; Age <12 yrs: 70.8%	RT-PCR	NR
Price 1988 (*12*)	Sierra Leone	1981-1985	Cohort	68 (NR)	Pregnant women; NR	Lassa IgG antibody titer ≥ 1:4 to ≥1:16, Lassa IgG antibody titer ≥1:256 and Lassa IgM antibody, or virus isolation	United States Army Medical Research and Development Command
Samuels 2020 (*13*)	Sierra Leone	Jan 2012 – Dec 2018	Cohort	57* (63.2%)	Children; Age<15yrs: 82%	ELISA for Lassa Ag, IgM and IgG	Fogarty International Center of the National Institutes of Health (NIH), National Institute of Allergy and Infectious Diseases, and U.S. Agency for International Development (USAID)
Shaffer 2014 (*1*,*2*)	Sierra Leone	2008-2012	Cohort	97* (37.1%)	Children and adults; Age<15 yrs: 70.1%	Positive Lassa virus Ag ELISA, IgM ELISA, or IgG ELISA	National Institute of Allergy and Infectious Diseases and Burroughs Wellcome Fund
Wauquier 2020 (*14*)	Sierra Leone	NR	Cohort	79 (39.2%)	Children and adults; Median: 22 (IQR: 14-30)	RT-PCR	French National Agency of Research (ANR-13-BSV-0004)

Abbreviations: Ag: antigen; ELISA: enzyme-linked immunosorbent assay; IFA: immunofluorescent-antibody assay; IQR: interquartile range; NR: not reported; RT-PCR: reverse transcription PCR.
*Confirmed cases only.
†Birch & Davis Associates and Sherikon Inc., US Army Medical Research and Development Command, unpub. data, https://media.tghn.org/medialibrary/2019/03/Responsive_Documents_of_Peter_Horby.pdf.pdf
‡M.-L. Orji et al., unpub. data, https://doi.org/10.20944/preprints202005.0269.v1.

Appendix Table 4. Summary of treatment regimens

Study	Ribavirin treatment regimen	No ribavirin treatment	Other case management
Ajayi 2013 (*4*)	NR	Supportive therapy	NR
Asogun 2012 (*5*)	NR	NR	NR
Buba 2018 (*6*)	NR	NR	NR
Dahmane 2014 (*7*,*8*)	Loading dose of 30 mg/kg, followed by 15 mg/kg QID from day 1 to 4 and 7.5 mg/kg TID from day 5 to 10	NR	Patients with malaria positive on testing received anti-malarial drugs, and antibiotics if clinically indicated
Ilori 2019 (*9*)	NR	NR	NR
IND 16666*	Regimen 2: IV Ribavirin followed by oral dose<br>Regimen 3: Ribavirin + plasma<br>Regimen 5: Ribavirin 25-30mg loading dose<br>Regimen 6: Ribavirin 34mg loading dose<br>Regimen 7: Ribavirin 33mg loading dose followed by 1/4 dose<br>Regimen 8: Ribavirin 33mg loading dose followed by 1/8 dose<br>Regimen 9: Ribavirin + prostacyclin	Regimen 1: No treatment<br>Regimen 10: no drugs were available	NR
Joseph 2019 (*10*)	NR	NR	Antipyretics
McCormick 1986 (*11*)	IV ribavirin (1): 2-g loading dose and 1 g QID for 4 days, reduced to 0.5 g TID for another 6 days<br>IV ribavirin (2): 2-g loading dose and 1 g QID for 4 days, reduced to 0.5 g TID for another 6 days with 1 unit (300ml) of convalescent plasma<br>Oral ribavirin: 2-g loading dose followed by 1 g QID for 10 days	NR	NR
Orji 2020†	NR	NR	NR
Price 1988 (*12*)	NR	NR	Chloroquine and broad-spectrum antibiotics until Lassa fever was confirmed
Samuels 2020 (*13*)	Loading dose of IV ribavirin 30 mg/kg with 24 hours of admission, and then maintenance dose as follow: 15 mg/kg every 6 hours for 4 days followed by 7.5 mg/kg every 8 hours for 5 days to complete 10 total days of therapy	Supportive care provided according to the WHO Integrated Management of Childhood Illnesses guidelines (prior 2017) or the WHO Emergency Triage Assessment and Treatment guidelines (*15*,*16*), which involved IV fluids, use of oxygen, nasogastric feeding, and catheterization, and treatment of comorbidities when necessary and available.	Broad spectrum antibiotics with either intravenous ceftriaxone or cefotaxime, depending on age; intravenous antimalarial medications if a rapid malaria test was positive; and blood transfusions for patients with anemia
Shaffer 2014 (*1*,*2*)	NR	NR	NR
Wauquier 2020 (*14*)	NR	NR	Antibiotics, antimalarials and other medicines (not specified)

Abbreviations: IV: intravenous; NR: not reported; QID: four times a day; TID: three times a day
*Birch & Davis Associates and Sherikon Inc., US Army Medical Research and Development Command, unpub. data, https://media.tghn.org/medialibrary/2019/03/Responsive_Documents_of_Peter_Horby.pdf.pdf
†M.-L. Orji et al., unpub. data, https://doi.org/10.20944/preprints202005.0269.v1.

Appendix Table 5. Case fatality rates and odd ratios for the effect of ribavirin compared with no ribavirin from mean and sensitivity analyses

Test	%Case fatality rate (death/total)		Odds ratio
	Ribavirin	No ribavirin	(95% CI)
Ag+ only (main analysis)	59.5% (44/74)	60.9% (14/23)	0.94 (0.36-2.46)
IgM+ only (IgM+ in serostatus 1 or 2)	44.8% (64/143)	41.9% (18/43)	1.13 (0.57-2.24)
IgM+ only (IgM+ in serostatus 1 and 2)	37.4% (34/91)	17.4% (4/23)	2.83 (0.89-9.03)
IgG+ only	21.4% (3/14)	100.0% (2/2)	*
Ag+ or IgM+ (IgM+ in serostatus 1 or 2)	44.8% (64/143)	41.9% (18/43)	1.13 (0.57-2.24)
Ag+ or IgM+ (IgM+ in serostatus 1 and 2)	44.8% (64/143)	41.9% (18/43)	1.13 (0.57-2.24)
Ag+ or IgG+	55.4% (46/83)	62.5% (15/24)	0.75 (0.29-1.90)
IgM+ or IgG+ positive (IgM+ in serostatus 1 or 2)	44.8% 64/143)	41.9% (18/43)	1.13 (0.57-2.24)
IgM+ or IgG+ positive (IgM+ in serostatus 1 and 2)	36.8% (35/95)	20.8% (5/24)	2.22 (0.76-6.46)
Ag+, IgM+ or IgG+	44.8% (64/143)	41.9% (18/43)	1.13 (0.57-2.24)
Ag- only (suspected cases)	30.1% (41/136)	27.6% (27/98)	1.13 (0.64-2.02)
IgM- only (IgM- in serostatus 1 and 2)	31.3% (21/67)	29.5% (23/78)	1.09 (0.54-2.22)
IgM- only (IgM- in serostatus 1 or 2)	42.9% (51/119)	37.8% (37/98)	1.24 (0.72-2.14)
IgG- only	41.8% (82/196)	32.8% (39/119)	1.48 (0.92-2.38)
Ag- or IgM- (IgM- in serostatus 1 and 2)	31.3% (21/67)	29.5% (23/78)	1.09 (0.54-2.22)
Ag- or IgM- (IgM- in serostatus 1 or 2)	31.3% (21/67)	29.5% (23/78)	1.09 (0.54-2.22)
Ag- or IgG-	30.7% (39/127)	26.8% (26/97)	1.21 (0.67-2.18)
IgM- or IgG- positive (IgM+ in serostatus 1 and 2	31.3% (21/67)	29.5% (23/78)	1.09 (0.54-2.22)
IgM- or IgG- positive (IgM+ in serostatus 1 or 2)	43.5% (60/115)	37.1% (36/97)	1.3 (0.75-2.27)
Ag-, IgM- or IgG-	31.3% (21/67)	29.5% (23/78)	1.09 (0.54-2.22)
All cases	40.5% (85/210)	33.9% (41/121)	1.33 (0.83-2.12)

* Not estimable
